# Increased DNMT1 Involvement in the Activation of LO2 Cell Death Induced by Silver Nanoparticles via Promoting TFEB-Dependent Autophagy

**DOI:** 10.3390/toxics11090751

**Published:** 2023-09-04

**Authors:** Jialong Chen, Dongyan Zheng, Ziwei Cai, Bohuan Zhong, Haiqiao Zhang, Zhijie Pan, Xiaoxuan Ling, Yali Han, Jinxue Meng, Huifang Li, Xiaobing Chen, He Zhang, Linhua Liu

**Affiliations:** 1 Department of Preventive Medicine, School of Public Health, Guangdong Medical University, Dongguan 523808, China; jialongc@gdmu.edu.cn; 2 Dongguan Key Laboratory of Environmental Medicine, School of Public Health, Guangdong Medical University, Dongguan 523808, China; zhengdongyan99@163.com (D.Z.); caiziwi98@163.com (Z.C.); 15019927095@163.com (B.Z.); zhanghaiqiao1992@163.com (H.Z.); 13650374217@163.com (Z.P.); xiaoxuanling@163.com (X.L.); h18565950156@163.com (Y.H.); mjx_11071005@163.com (J.M.); lihuifang09@163.com (H.L.); chenxiaobing2021@163.com (X.C.)

**Keywords:** silver nanoparticles, autophagy, DNMT1, hepatocyte injury, cell death

## Abstract

The accumulation of exogenous silver nanoparticles (AgNPs) will terminally bring about liver injury, including cell death, where DNA methylation tends to be a crucial epigenetic modulator. The change in the cell autophagy level verified to be closely associated with hepatocyte death has been followed with wide interest. But the molecular toxicological mechanisms of AgNPs in relation to DNA methylation, autophagy, and cell death remain inconclusive. To address the issue above, in LO2 cells treated with increasing concentrations of AgNPs (0, 5, 10, and 20 μg/mL), a cell cytotoxicity assay was performed to analyze the level of cell death, which also helped to choose an optimal concentration for next experiments. An immunofluorescence assay was used to determine the autophagic flux as well as TFEB translocation, with qRT-PCR and western blot being used to analyze the expression level of autophagy-related genes and proteins. According to our findings, in the determination of cell viability, 20 μg/mL (AgNPs) was adopted as the best working concentration. LO2 cell death, autophagy, and TFEB nuclear translocation were induced by AgNPs, which could be inhibited by lysosome inhibitor chloroquine (CQ) or siRNA specific for TFEB. Moreover, AgNP exposure led to DNA hypermethylation, with DNMT1 taking part mainly, which could be obviously prevented by 5-Aza-2′-deoxycytidine (5-AzaC) or trichostatin A (TSA) treatment or DNMT1 knockout in LO2 cells. Our studies suggest that through TFEB-dependent cell autophagy, increased DNMT1 may facilitate cell death induced by AgNPs.

## 1. Introduction

With the rapid progression of nanotechnology, nanomaterials and nano-products are extensively produced and used in various fields, leading to an increasing potential for human exposure [1]. Silver nanoparticles (AgNPs) serve as one of the members of nanomaterials. Due to their unique physicochemical properties, AgNPs play indispensable roles in widespread aspects, including food packaging, medical products and devices, wound healing, dental materials, bone healing, and so on [2], and such a wide application has also raised much concern regarding the toxicity and negative impacts of AgNPs on human health [3]. Humans can be exposed to AgNPs through several pathways, such as ingestion [3], inhalation [4], and injection [5]. Early studies have shown that AgNP levels in the air ranged from 5 to 289 µg/m^3^ [6]. The absorbed AgNPs mainly accumulate in the liver and can cause liver injury [7]. However, studies on the mechanisms of AgNP-induced hepatocellular injury have not been completely characterized yet. Present studies have demonstrated that AgNPs exert adverse influence by producing reactive oxygen species (ROS), inducing excessive free radicals, impairing DNA and that results in mitochondria damage, altering cell cycle and cell autophagy and so on, which contributes to hepatocellular cytotoxicity [8]. In-depth elucidation on the molecular mechanisms of AgNPs is of great significance in preventing and relieving liver damage, especially cell death.

Studies have shown that hepatocyte death caused by AgNPs accounted partly for the disorder of cell autophagy [9]. With the regulation of autophagy-related genes (ATGs), cell autophagy refers to the process of cells using lysosomes to degrade damaged organelles as well as macromolecules, which are essential for maintaining the stability of liver cells, tissues, and organs. Both excessive and low degrees of autophagy may contribute to hepatocyte damage, causing autophagic, apoptotic, necrotic, or other kinds of cell death. For example, inhibiting autophagy could effectively make promotion to the occurrence of apoptotic death to benefit the treatment of hepatocellular carcinoma [10]. Intraperitoneally injected with AgNPs in rats caused hepatocyte apoptotic death due to autophagy downregulation, causing liver function impairing eventually [11]. Nevertheless, it remains inconclusive whether AgNPs induce cell death and the type of death in normal human liver cells. More than 40 ATGs, which consist of ATG1, ATG2, ATG3, ATG4, ATG7, microtubule-associated protein1 light chain 3 (MAP1LC3, LC3), Beclin1, a mechanistic target of rapamycin kinase (M-TOR), B-cell lymphoma-2 (Bcl2), sequestosome 1 (SQSTM1, P62), tumor protein p53 (P53), and phosphatidylinositol 3-kinase VPS34 (VPS34) have so far been identified in mammals like yeast, man, and mice, and play an important role in regulating the formation of autophagosome and the combination of autophagosomes and lysosomes. Moreover, transcription factor EB (TFEB), a capital member for lysosomal biogenesis and autophagy adjustment, is also considered to regulate autophagy permanently [12]. Therefore, we also made an analysis of the function of TFEB in AgNP-induced LO2 cell autophagy.

DNA methylation, a kind of critical epigenetic modification, is reversible. It occurs under the catalysis of DNA methyltransferases (DNMTs), which mainly include DNMT1, DNMT3a and DNMT3b, while DNA demethylation is initiated by ten to eleven translocations (TETs), primarily containing TET1, TET2, and TET3. DNA methylation partakes in vital biological processes such as gene expression modulation, gene imprinting, transposon silencing, X chromosome inactivation, and so on [13]. Recent evidence has shown that DNA methylation was maladjusted in some animal disease models treated with AgNPs, like non-alcoholic fatty liver disease [5]. Furthermore, emerging studies have also put forward the point that DNA methylation was an intimate participant in the process of cell autophagy [14]. For instance, the promotion of cell autophagy in triple-negative breast cancer was associated with DNA hypermethylation due to an increase in DNMT1 [15]. DNMT1 hypermethylation enhanced the level of autophagy in homocysteine-treated hepatocytes [16]. Therefore, we made an assumption that DNA methylation modification is involved in regulating the LO2 cell autophagy.

In our present study, with the aim of investigating the above assumption, the level of autophagy and TFEB nuclear translocation in LO2 cells exposed to AgNPs were found to be upregulated and further suppressed under 5-AzaC or TSA treatment. AgNP exposure also aggravated LO2 cell death. Moreover, under the same conditions, the expression of DNMT1 was increased, and its knockout could downregulate cell autophagy markedly. Therefore, increased DNMT1 might be involved in the activation of LO2 cell death induced by AgNPs via promoting TFEB-dependent autophagy.

## 2. Materials and Methods

### 2.1. AgNPs

The spherical powder of AgNPs was obtained from LHNANO, Shanghai, China (LH-Ag-20) with an average diameter of 50 nm, high density (99.9%), and excellent liquidity. Also, according to the supplied information, the specific surface area and density were 24.9 m^2^/g and 1.58 g/cm^3^, respectively. Full details, including transmission electron microscopy (TEM) images, are shown at https://china.guidechem.com/trade/pdetail18911950.html (accessed on 31 August 2023).

### 2.2. Cell Culture, Chemical Treatment, and Transfection

Immortalized human embryonic liver cell lines LO2, serving as feasible sources of the liver support system, are used in the study of the molecular mechanisms of liver diseases for their established applications in evaluating the hepatotoxicity of exogenous compounds [17]. LO2 cell lines were acquired from the American Type Culture Collection (ATCC, Manassas, VA, USA) and maintained in an RPMI 1640 medium (Gibco, New York, NY, USA) supplemented with 10% fetal bovine serum (FBS, Biological Industries, Kibbutz Beit Haemek, Israel) at 37 °C with 5% CO_2_. The culture dish was purchased from Sorfa (Beijing, China). AgNPs were dissolved in a 1640 medium and instantly exerted on cells (0, 5, 10, and 20 μg/mL) for 24 h. Further, with the reference of early research [6,18] and the determination of our cell viability assay, 20 μg/mL was adopted as the dose concentration of AgNPs for the next experiments, which ensured that LO2 cell responses to AgNP exposure could be observed and the influence of dead cells in the samples could be minimized. Moreover, the above exponentially grown cells were individually treated by 5-Aza-2′-deoxycytidine (5-AZA, 2353-33-5, Sigma, St Louis, MO, USA) at 5 μmol/L, trichostatin A (TSA, 58880-19-6, Sigma, St Louis, MO, USA) at 0.2 μmol/L for 24 h, or chloroquine (CQ, C6628, Sigma, St Louis, MO, USA) at 10 μmol/L for 4 h. Then, the cells were harvested for total RNA and protein, respectively. Additionally, according to the manufacturer’s instructions, the knockdown of TFEB was performed with the use of a Lipofectamine 3000 reagent (Invitrogen, Carlsbad, CA, USA). The experiments were repeated three times.

### 2.3. Cell Cytotoxicity Assay

The cell cytotoxicity assay was performed with a Cell Counting Kit-8 (CCK-8, Dojindo, Tokyo, Japan) and a lactate dehydrogenase (LDH) Assay Kit (Beyotime, Shanghai China). Firstly, at a density of 10,000 cells/well (100 μL/well), LO2 cells were seeded in a 96-well plate in triplicate for 24 h. Secondly, the cells were dealt with AgNPs or CQ at a proper concentration for 0, 6, 12, or 24 h. After exposure, a CCK-8 solution or an LDH Release Reagent (100 μL/mL medium) was added to the plate. For the CCK-8 assay, the plate was kept in a dark condition at 37 °C for 2 h. For the detection of extracellular LDH, the plate was centrifuged at 400× *g* for 5 min, and then the supernatant from each well was transferred into a new 96-well plate (120 μL/well). A microplate reader was used to analyze the absorbance values at 450 or 490 nm.

### 2.4. Knockout of DNMT1 by CRISPR/Cas9

Initially, DNMT1 small-guide RNA (sgRNA) was inserted into the lenti-CRISPR-vector (GENE). The sequences of DNMT1 sgRNA were 5′-TCCTGAGGTTTCCGTTTGGCA-3′, 5′-CTTGATGGACTCATCCGATT-3′, and 5′-TTTCCAAACCTCGCACGCCC-3′. Then, CRISPR/Cas9 lentivirus was transfected into LO2 cells. After puromycin selection, stable CRISPR/Cas9 cell lines were established, and these were utilized to construct DNMT1-knockout cells after neomycin G418 screening. Without a sgRNA sequence, the control cells were transfected with lenti-CRISPR/Cas9 constructs.

### 2.5. Reverse Transcription–Quantitative Polymerase Chain Reaction (qRT-PCR)

Total RNA was isolated by measuring the mRNA production of associated genes with the use of TRIzol (Invitrogen, Carlsbad, CA, USA) according to the manufacturer’s instructions. Then, cDNA was initially synthesized with 1 μg RNA using a RevertAid First Strand cDNA Synthesis Kit (Thermo Fisher Science, Carlsbad, CA, USA). Next, qRT-PCR was carried out utilizing the FastStart Universal SYBR Green Master kit (Roche, Mannheim, Germany) and an ABI7500 PCR instrument (Applied Biosystems, Thermo Fisher Scientific, Waltham, MA, USA). Primers were as follows: DNMT1 (forward: 5′-TTGGAGAACGGTGCTCATGCTTA-3′ and reverse: 5′-CATCTGCCATTCCCACTCTACGG-3′); DNMT3A (forward: 5′-TATTGATGAGCGCACAAGAGAGC-3′ and reverse: 5′-GGGTGTTCCAGGGTAACATTGAG-3′); DNMT3b (forward: 5′-GGCAAGTTCTCCGAGGTCTCTG-3′ and reverse: 5′-TGGTACATGGCTTTTCGATAGGA-3′); LC3 (forward: 5′-GCTTGCAGCTCAATGCTAAC-3′ and reverse: 5′-CCTGCGAGGCATAAACCATGTA-3′); Beclin1 (forward: 5′-CTGGACACTCAGCTCAACGTCA-3′ and reverse: 5′-CTCTAGTGCCAGCTCCTTTAGC-3′); and GAPDH (forward: 5′-GGAGTCAACGGATTTGGTCGTATTG-3′ and reverse: 5′-TCTCGCTCCTGGAAGATGGTGAT-3′). The conditions of qRT-PCR were an initial cycle at 95 °C for 10 min, 40 cycles at 95 °C for 15 s, and 60 °C for 40 s. The method of 2^−ΔΔCt^ was used to determine the comparative quantification of the targets with glyceraldehyde-3-phosphate dehydrogenase (GAPDH) as the inherent control.

### 2.6. Western Blot Analysis

The concentration of total cellular proteins was measured by a bicinchoninic acid (BCA) Protein Assay Kit (Kangwei Technology, Beijing, China) after total proteins were extracted with the use of a cell lysis buffer that was purchased from KeyGEN (KGP701, Nanjing, China), based on the manufacturer’s direction. The supernatant was separated by 10% SDS-PAGE gels (20 μg/lane). The above proteins were transferred to polyvinylidene fluoride (PVDF) membranes (Millipore, Billerica, MA, USA) that were blocked with 3% bovine serum albumin (BSA) in TBS with 0.1% Tween-20 (TBST) for 60 min at room temperature (RT). After that, the membranes were incubated with primary antibodies of α-tubulin (1:5000, 66031-1-Ig, Proteintech, Chicago, IL, USA), LC3 (1:1000, 14600-1-AP, Proteintech, Chicago, IL, USA), p62 (1:5000, 18420-1-AP, Proteintech, Chicago, IL, USA), GAPDH (1:5000, 60004-1-Ig, Proteintech, Chicago, IL, USA), DNMT1 (1:1000, #5032, Cell Signaling Technology, Danvers, MA, USA), DNMT3a (1:1000, #32578, Cell Signaling Technology, Danvers, MA, USA), DNMT3b (1:1000, #57868, Cell Signaling Technology, Danvers, MA, USA), and Caspase 3 (1:2000, 19677-1-AP, Proteintech, Chicago, IL, USA) at 4 °C overnight. Subsequently, the membranes were incubated with a proper secondary antibody (1:5000, Bioworld, Nanjing, China) at RT for 1 h. GAPDH or α-tubulin was used as the internal control. The aimed proteins were visualized with a chemiluminescence kit (BOSTER, Shanghai, China), and ImageJ software was used to make the intensity analysis [19]. 

### 2.7. Immunofluorescence Assay

LO2 cells seeded in a 12-well plate were fixed with 4% paraformaldehyde for 15 min at RT, washed with PBS three times, and permeabilized with frozen methanol for 10 min at −20 °C. After blocking with 5% BSA for 30 min, cells were incubated with primary antibodies targeting LC3 (1:250) or TFEB (1:50, 13372-1-AP, Proteintech, Chicago, IL, USA) in NCM Universal Antibody Diluent (New Cell & Molecular Biotech, Suzhou, China) at 4 °C overnight after being washed for three times. Next, cells were incubated with anti-rabbit Alexa Fluor 594 antibodies (1:100, Thermo Fisher Scientific, Waltham, MA, USA) in 5% BSA at RT for 1 h and stained with DAPI. Olympus FV1000 Confocal Laser Scanning Microscopy was used to observe the cells.

### 2.8. Assay for Detecting Lysosomal Activity

LO2 cells were incubated with LysoTracker Red (#KGMP006, KeyGen, Nanjing, China) at 50 nM in a medium at 37 °C for 25 min after treatment of AgNPs. Subsequently, PBS was utilized for washing the cells immediately for unbound LysoTracker removal. Cells could be visualized under the Olympus FV1000 Confocal Laser Scanning Microscopy. The lysosome activity was measured by fluorescence intensity.

### 2.9. Statistical Analysis

The quantitative data of three separate experiments are presented as the mean ± standard deviation (SD). The differences between the samples were evaluated using a double-sided Student’s *t*-test. SPSS software (version 25.0) was used for statistical analysis, with *p* < 0.05 considered significant.

## 3. Results

### 3.1. AgNP Treatment Decreased Cell Viability and Increased LDH Release in LO2 Cells

**Figure 1:**
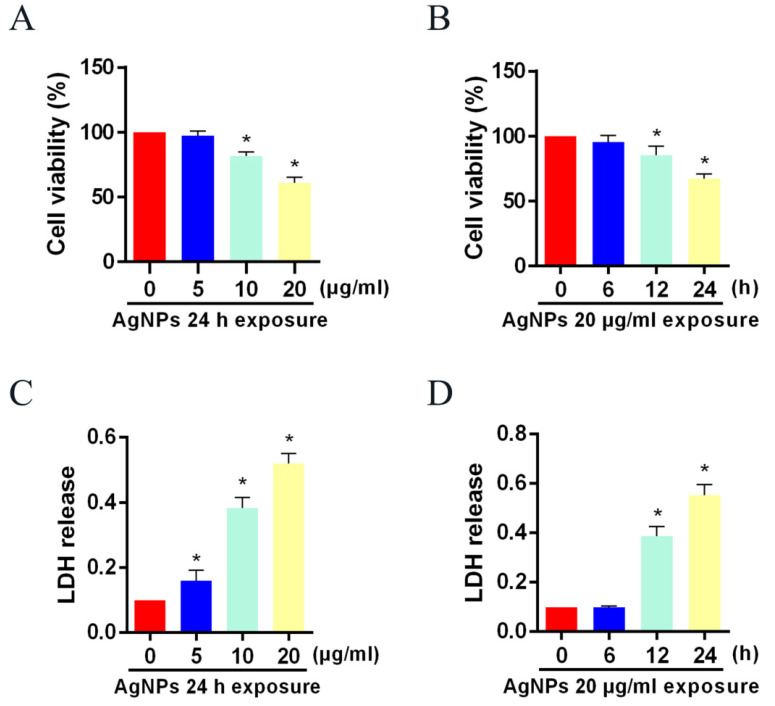
Toxicity of AgNPs to LO2 cells. Cell viability and LDH release level of LO2 cells after (**A**,**C**) 24 h of AgNP (0, 5, 10, 20 μg/mL) treatments and (**B**,**D**) different time courses of AgNP (20 μg/mL) treatments, respectively. (* *p* < 0.05 vs. the 0 μg/mL or 0 h AgNPs group.) AgNPs, silver nanoparticles; LDH, lactic dehydrogenase.

CCK-8 and LDH assays were first used to determine the cytotoxicity of AgNPs on LO2 cells. A noticeable cell viability decrease, as well as an LDH release increase, were found after 10 or 20 μg/mL of AgNP treatment for 24 h compared with the control group (Figure 1A–C). Additionally, 24 h AgNP (20 μg/mL) exposure decreased LO2 cell viability and increased LDH release. (Figure 1B–D). For this reason, LO2 cells treated with increasing concentrations of AgNPs for 24 h were chosen for further study.

### 3.2. AgNPs Activated Autophagy in LO2 Cells

**Figure 2:**
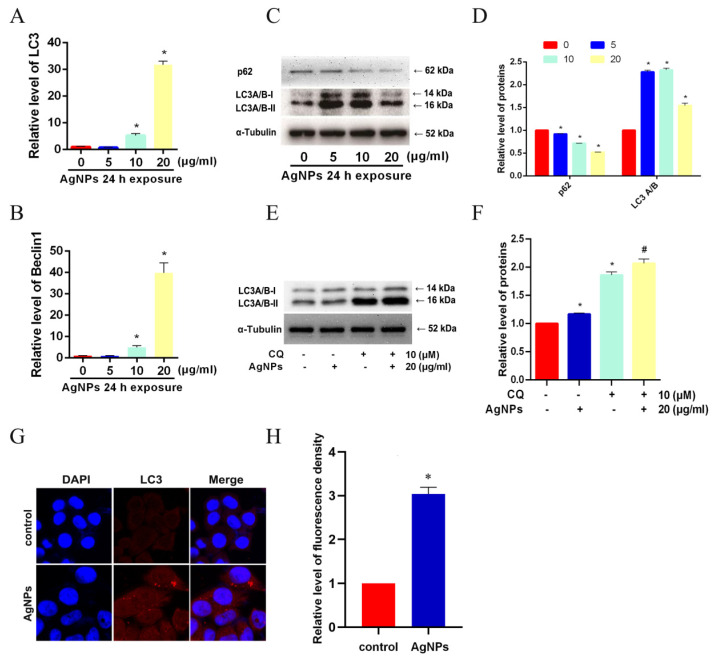
Induction of autophagy was found in LO2 cells treated with AgNPs. (**A**,**B**) qRT-PCR was used to detect the mRNA expression levels of LC3 and Beclin1 in LO2 cells dealt with 24 h AgNPs (0, 5, 10, 20 μg/mL). An immunoblot assay was used to measure the protein expression level of (**C**,**D**) p62 and LC3 after 24 h of AgNP (0, 5, 10, 20 μg/mL) treatments, as well as (**E**,**F**) LC3 upon AgNP-only treatment or AgNP-CQ co-treatment. (**G**,**H**) Punctate LC3 signals of LO2 cells treated with 24 h of AgNP (20 μg/mL) were measured via IFC analysis. (* *p* < 0.05 vs. the 0 μg/mL AgNP group or the control group; ^#^ *p* < 0.05 vs. the AgNPs treatment group.) AgNPs, silver nanoparticles; CQ, chloroquine; IFC, immunofluorescence.

To determine if AgNPs could induce cell autophagy, qRT-PCR and western blot assays were used to analyze the expression level of autophagy-related genes and proteins in LO2 cells. Results showed that mRNA expression levels of both LC3 and Beclin1 were upregulated after the treatment of AgNPs at 10 or 20 μg/mL (Figure 2A,B). In Figure 2C,D, LC3 A/B-II increased and p62 decreased in comparison with the control group, indicating the activation of autophagic flux in LO2 cells. Further, the addition of a well-characterized lysosome inhibitor CQ (at 10 μM for 4 h) [20] and immunofluorescence assay provided evidence against the view that the increase in LC3 A/B-II resulted from lysosome dysfunction caused by AgNPs. We found that CQ-only treatment could markedly increase the expression level of LC3 A/B-II protein (Figure 2E,F). Under the addition of AgNPs, the LC3 A/B-II protein level further increased due to the effect of CQ. In addition, the increase in punctate LC3 signals was found in LO2 cells treated with AgNPs, indicating the formation of autophagosomes (Figure 2G,H).

### 3.3. AgNPs Induced TFEB-Dependent Autophagy

**Figure 3:**
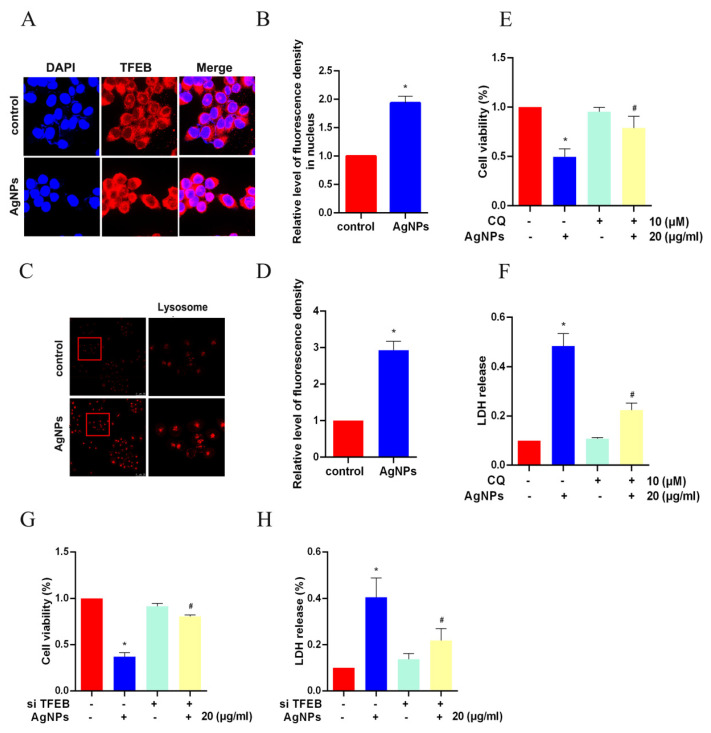
AgNPs induced TFEB-dependent cell autophagy. (**A**,**B**) LO2 cells were treated with AgNPs (20 μg/mL) for 24 h and immunostained for TFEB (red). The nuclei were stained with DAPI (blue). (**C**,**D**) LysoTracker dyes (red) were used after the treatment of AgNPs (20 μg/mL) for 24 h. (**E**,**F**) Cell viability and the LDH release level of LO2 cells upon AgNP-only treatment or AgNP-CQ co-treatment. (**G**,**H**) Cell viability and the LDH release level were analyzed in LO2 cells transfected with siRNA specific for TFEB (si TFEB) and treated with AgNPs (20 μg/mL) for an additional 24 h. (* *p* < 0.05 vs. the control group; ^#^ *p* < 0.05 vs. the AgNPs treatment group.) AgNPs, silver nanoparticles; TFEB, transcription factor EB; DAPI, 4′,6-diamidino-2-phenylindole; LDH, lactic dehydrogenase; CQ, chloroquine.

We further made an analysis of the regulator of cell autophagy. Nuclear transcription factor EB (TFEB) (Figure 3A,B), as well as the number of lysosomes (Figure 3C,D), were upregulated in LO2 cells with the addition of AgNPs, indicating that lysosomal biogenesis may be improved via TFEB nuclear translocation. Additionally, cell viability and LDH release levels could be partly restored by the use of autophagy inhibitor CQ (Figure 3E,F). To confirm the effect of TFEB, siRNA specific for TFEB was transfected into LO2 cells. As was shown in Figure 3G,H, si TFEB could recover the decrease in cell viability and increase in LDH release level caused by AgNPs. Consequently, we concluded that AgNPs may induce LO2 cell autophagy and lysosomal biogenesis by activating the TFEB nuclear translocation.

### 3.4. DNMTs Were Involved in the Autophagy after AgNP Exposure 

**Figure 4:**
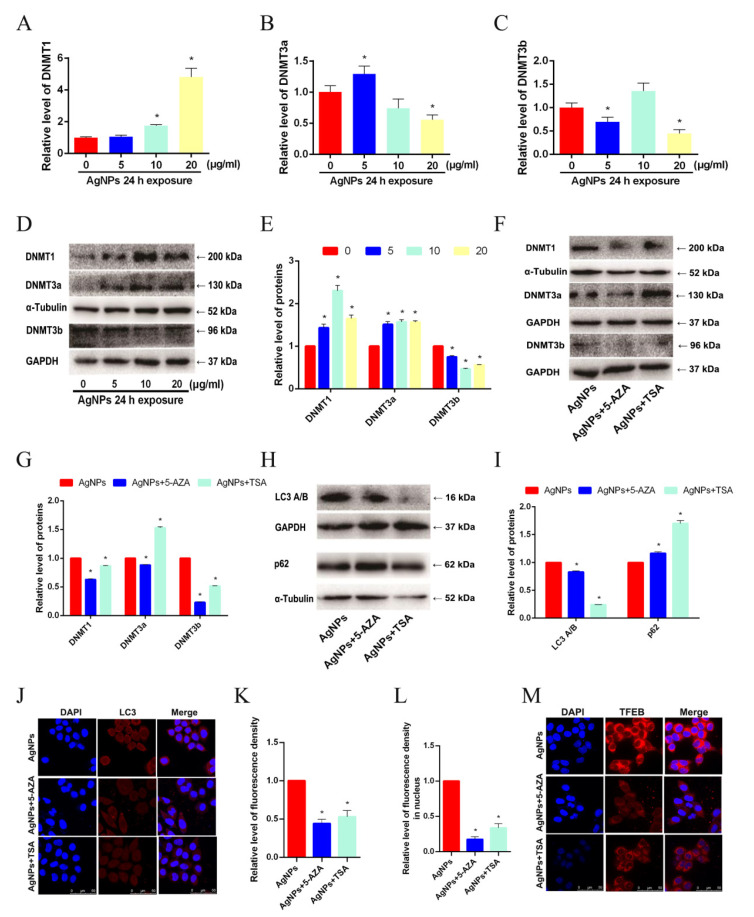
DNMTs were involved in autophagy after AgNP exposure. (**A**–**C**) qRT-PCR was used to detect the mRNA expression levels of DNMTs, including DNMT1, DNMT3a, and DNMT3b (the same below), in LO2 cells dealt with 24 h of AgNP (0, 5, 10, 20 μg/mL). (**D**,**E**) The protein levels of DNMTs in LO2 cells after 24 h of AgNP (0, 5, 10, 20 μg/mL) treatments. A western blot was applied to detect the expression of (**F**,**G**) DNMTs, as well as (**H**,**I**) p62 and LC3, upon AgNP-5-AZA or AgNP-TSA co-treatment. (**J**,**K**) Punctate LC3 signals of LO2 cells upon AgNP-5-AZA or AgNP-TSA co-treatment were measured via IFC analysis. (**L**,**M**) LO2 cells were treated with AgNPs (20 μg/mL), as well as 5-AZA or TSA, for 24 h and immunostained for TFEB (red). The nuclei were stained with DAPI (blue). (* *p* < 0.05 vs. the control group). DNMTs, DNA methyltransferases; AgNPs, silver nanoparticles; 5-AZA, 5-Aza-2′-Deoxycytidine; TSA, Trichostatin A; IFC, immunofluorescence; TFEB, transcription factor EB; DAPI, 4′,6-diamidino-2-phenylindole.

DNA methyltransferases (DNMTs) are crucial for DNA methylation and transcriptional control in the genome, but their involvement in the AgNP-induced autophagy process is still unclear. To verify the involvement of DNMTs in autophagy, qRT-PCR and western blot were used. AgNPs upregulated the mRNA levels of DNMT1 and DNMT3a, and the western blot results coincided with qRT-PCR data, while there was no observable increase in DNMT3b (Figure 4A–E). In addition, based on a previous study [21], an inhibitor of DNMTs 5-Aza-2′-Deoxycytidine (5-AZA), and an inhibitor of histone deacetylase (HDAC) Trichostatin A (TSA), were applied to make sure that DNMT was involved in the autophagy. The 5-AZA forms a covalent adduct with DNMT1 to inhibit its activity [22], and TSA can also influence the level of methylation by influencing the interaction between histone acetylation and methylation binding proteins [23]. In Figure 4F,G, 5-AZA or TSA could downregulate DNMT1 and DNMT3b, but not DNMT3a. An LC3 A/B-II decrease and p62 increase were found after the deal of 5-AZA or TSA in comparison with the control group (Figure 4H,I), indicating the inhibition of autophagic flux in LO2 cells. Additionally, both 5-AZA and TSA reduced the autophagosome formation (Figure 4J,K), as well as TFEB nuclear translocation (Figure 4L,M), in LO2 cells treated with AgNPs. Substantially, DNMTs, especially DNMT1, may become involved in LO2 cell autophagy and lysosomal biogenesis caused by AgNPs through inhibiting the TFEB nuclear translocation, which could be effectively decreased by the suppression of DNA methylation.

### 3.5. DNMT1 Knockout Inhibited TFEB-Dependent Autophagic Cell Death after AgNP Exposure

**Figure 5:**
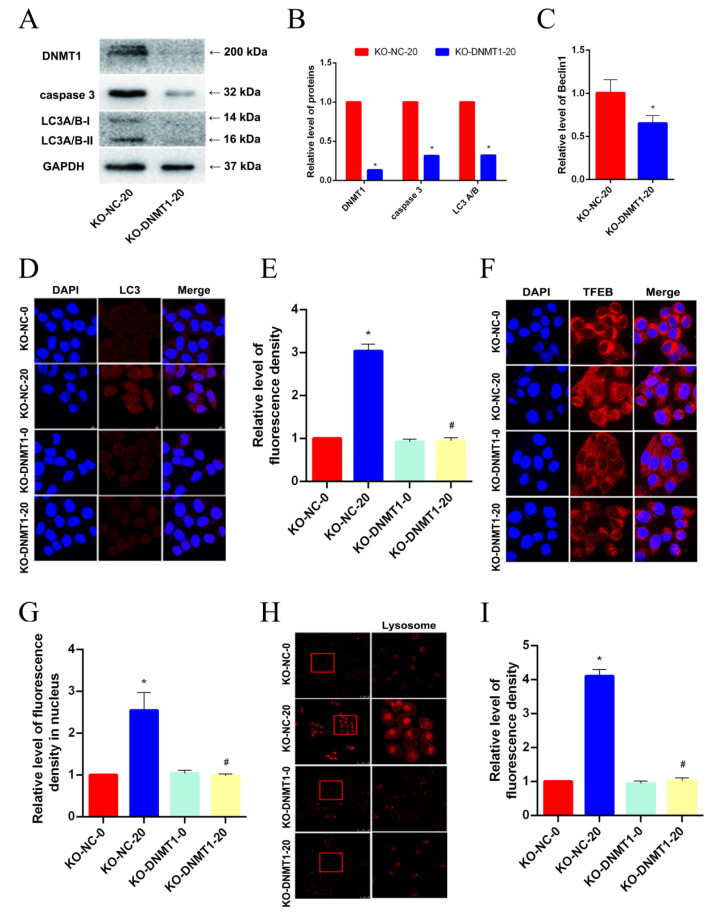
DNMT1 knockout inhibits TFEB-dependent autophagic cell death after AgNP exposure. (**A**–**C**) Western blot and qRT-PCR were used to detect the protein expression levels of DNMT1, caspase 3, LC3 A/B and the mRNA level of Beclin1 in AgNP (20 μg/mL)-treated LO2 cells with or without DNMT1 knockout. (**D**,**E**) Punctate LC3 signals of LO2 cells upon AgNP (20 μg/mL) treatment with or without DNMT1 knockout were measured via IFC analysis. (**F**,**G**) LO2 cells with or without DNMT1 knockout were treated with AgNPs (20 μg/mL) for 24 h and immunostained for TFEB (red). The nuclei were stained with DAPI (blue). (**H**,**I**) LysoTracker dyes (red) were used after the treatment of AgNPs (20 μg/mL) for 24 h. (* *p* < 0.05 vs. the KO-NC-0 group; ^#^ *p* < 0.05 vs. the KO-NC-20 group.) TFEB, transcription factor EB; AgNPs, silver nanoparticles; IFC, immunofluorescence; DAPI, 4′,6-diamidino-2-phenylindole; KO-NC-0 group, LO2 cells dealt without AgNPs; KO-NC-20 group, LO2 cells dealt with AgNPs (20 μg/mL); KO-DNMT1-0 group, DNMT1 knocking out LO2 cells dealt without AgNPs; KO-DNMT1-20 group, DNMT1 knocking out LO2 cells dealt with AgNPs (20 μg/mL).

To further validate whether DNMT1 regulates the level of cell autophagy, we knocked out DNMT1 in LO2 cells by employing CRISPR/Cas9 dual vector lentiviral technology. In Figure 5A,B, the knockout effect was qualified. The protein levels of Caspase3 and LC3A/B-II definitely decreased for the knockout of DNMT1. The mRNA level of Beclin1 was also downregulated in AgNP-treated LO2 cells when the DNMT1 was exhausted, indicating that DNMT1 controlled the beginning of autophagy induced by AgNPs (Figure 5C). In addition, DNMT1 deletion reduced the autophagosome formation (Figure 5D,E), as well as TFEB nuclear translocation (Figure 5F,G), in LO2 cells treated with AgNPs. After AgNP exposure, the number of lysosomes reduced in DNMT1-knocked out LO2 cells,, in comparison with other groups (Figure 5H,I). Consequently, we inferred that DNMT1 knockout inhibited TFEB-mediated autophagy led by AgNPs.

## 4. Discussion

Recently, the toxicity of silver nanoparticles (AgNPs), especially cytotoxicity, has raised extensive interest [2,3]. Through respiratory tract inhalation, skin contact, and other channels, AgNPs could enter and then impair our body easily, partly by inducing autophagy and cell death. For example, Guangzhe Zheng [24] revealed that ferroptosis in macrophages could be triggered by AgNPs. Xiaoru Chang [25] paid attention to the neurotoxicity of AgNPs and concluded that the occurrence of autophagy and apoptosis could account for AgNP-induced cytotoxicity of mouse hippocampal neuronal cells. Significantly, the absorbed AgNPs mainly accumulate in the liver and then injure the organ [7], while the problem of the concrete mechanism of hepatotoxicity being caused by AgNPs still needs to be solved. For this reason, relevant exploration was conducted. We found that AgNP treatment may stimulate LO2 cell death by the TFEB–autophagy pathway, where DNMT1 played a vital regulatory role.

Serving as the major intracellular degradation system, cell autophagy refers to the process of the lysosome receiving and degrading cytoplasmic materials spontaneously to make self-renewal. On the one hand, autophagy has been regarded as a self-protection mechanism of cells, playing a key role in their growth, preventing metabolic stress and oxidative damage, maintaining intracellular homeostasis, synthesis, degradation, and recycling cellular products [26,27]. Mondoa activated autophagy by inhibiting autophagy-negative regulatory factors, such as Rubicon, preventing cell aging [28]. The activation of nuclear factor erythroid 2-related factor 2 contributed to cell autophagy, which inhibited ferroptosis and protected against liver injury [29]. On the other hand, metabolic stress, abnormal degradation, and even cell death may be caused by autophagy dysregulation [30]. Yanan Zhao [31] demonstrated that the accumulation of liver lipids induced by Pb was led by autophagy disorder through the SIRT1/mTOR pathway. Autophagic flux improvement could also preserve hepatocytes that relieved liver damage in acute-on-chronic liver failure [32]. Our research found that the administration of AgNPs in LO2 cells induced cell autophagy by enhancing the expression level of autophagy-related genes and proteins like LC3, p62, and Beclin1. Moreover, cell death activation was also found due to the great deal of AgNPs, which did not entirely chime with Tin Yan Wong’s findings [18]. In his study, AgNPs promoted DNA repair, as well as autophagy, avoiding LO2 cell death. It was suggested that autophagy showed a protective role in LO2 cells but failed to keep them away from the final death, the type of which may be autophagy-dependent cell death [33], apoptosis [25], ferroptosis [24], etc. Notably, we also discovered a change in the Beclin1 mRNA level, which was recently confirmed to be related to the occurrence of cell apoptosis [34]. Furthermore, chloroquine (CQ) was found to inhibit LO2 cell death and autophagy caused by AgNPs effectively, which suggested that AgNPs may result in an enhancement in the source of autophagy. CQ prevents autophagy by inhibiting the degradation of autophagosomes by lysosomes [20,35]. An abnormal degradation of autophagosomes due to AgNP-induced incomplete lysosomal dysfunction may also result in the increase in LC3 A/B-II. Therefore, drugs like 3-Methyladenine (3-MA), inhibiting the formation of autophagic precursors, are needed to further confirm the above speculation directly. In addition, further analysis to make sure the type of AgNP caused normal hepatocyte death and the concrete regulatory mechanism remain indispensable. 

Transcription factor EB (TFEB) helps to regulate the lysosomal function and the level of cell autophagy. With the nuclear translocation of TFEB, the expression of genes related to autophagy and lysosomal biogenesis increased positively, thereby promoting the formation of autophagosomes and the fusion of autophagosomes and lysosomes, as well as the degradation of autophagic substrate. Additionally, TFEB dysfunction has been demonstrated to be associated with the development of some diseases [36,37]. A study indicated that the level of autophagy was improved and liver damage was attenuated in chronic and acute liver failure, because of the increased TFEB nuclear translocation [36]. Cell pyroptosis led by ZnO nanoparticles, another popular nanomaterial, could be inhibited by autophagy and lysosomal biogenesis enhancement mediated by TFEB [38]. Nevertheless, it is imprecise whether TFEB is involved in regulating the liver injury induced by exogenous AgNP accumulation in the liver, which is gap that our study could fill. In our study, AgNPs accelerated lysosomal biogenesis and autophagy via facilitating TFEB nuclear translocation, exacerbating hepatocyte death, whereas the interference of TFEB restored and protected cells from dying dramatically.

Previous studies have confirmed that AgNP exposure in the liver may lead to DNA damage and epigenetic changes [5]. DNA methylation, one of the most extensively studied epigenetic modifications, is of great importance in maintaining the stability of the genome and normal growth and development [39]. In general, DNA methylation regulates gene expression by affecting the conformation of DNA molecules, imposing a steric hindrance effect, facilitating protein specificity, and changing transcriptional levels [39]. As the most important enzyme for DNA methylation regulation, DNMT-methyltransferases 1 (DNMT1) can use semimethylated DNA chains as templates to facilitate new DNA chain methylation [40] and functions in maintaining the DNA methylation pattern of the existing CpG island. DNMT1 has been validated as a vital regulatory effect on cell autophagy, and that affects the development of diseases [41]. In our research, it was proven that DNMT1 played an important role in monitoring the TFEB-dependent cell autophagy caused by AgNPs. DNMT1 knockout in LO2 cells led to a decrease in the TFEB level and nuclear translocation level, which downregulated autophagy, which suggested that in normal hepatocytes, AgNPs may have triggered cell death by promoting the TFEB-dependent autophagy mediated by DNMT1 hypermethylation. However, Anning Yang [42] analyzed and discovered that homocysteine could accelerate TFEB-dependent autophagy by DNA hypomethylation mediated by DNMT3b, so there was a difference in the mechanism of cell autophagy compared with our study. He further identified that homocysteine reduced CpG island methylation of the TFEB promoter region by DNMT3b and affected autophagy in hepatocytes. Further investigation is warranted to decipher the regulatory mechanism and region of DNA methylation on TFEB expression.

It is essential to take effective measures to reduce the toxicity of AgNPs, which could be achieved well by AgNP encapsulation with reference to the published works [43,44]. Jung-Hee Kim [45] also discovered that autophagy was not activated by encapsulated nanoparticles in human embryonic kidney cells. But relevant analysis is needed to clarify whether AgNP encapsulation alleviated its toxicity through the inhibition of cell autophagy. In addition, treatment targeting DNMT1 to reduce the toxic effect of AgNPs on normal liver cells may be a good strategy as well. In conclusion, our research found that AgNPs upregulated autophagy in LO2 cells by increasing the level of TFEB, as well as its nuclear translocation, activating cell death. Our study will provide the basis for the evaluation of the hepatotoxicity of AgNPs. Future studies should evaluate if in vitro toxicity observed in this study would translate into in vivo toxicity using animal models of liver injury.

## Data Availability

All data generated in this work are presented as graphs and tables and can be provided by the corresponding author upon reasonable request.
